# Supplementary material for: Smartphone-Integrated User-Friendly Electrochemical Biosensor Based on Optimized Aptamer Specific to SARS-CoV-2 S1 Protein
Source: Sensors (Basel). 2025 Oct 25;25(21):6579. doi: 10.3390/s25216579 (PMC12610881; doi:10.3390/s25216579)
Supplement: Supplementary file 1 [file sensors-25-06579-s001.zip › sensors-3926331-supplementary.pdf]

Supplementary Materials

Smartphone-Integrated User-Friendly Electrochemical Biosensor Based on Optimized Aptamer Specific to SARS-CoV-2 S1 Protein

Arzum Erdem*, Huseyin Senturk and Esma Yildiz

Department of Analytical Chemistry, Faculty of Pharmacy, Ege University, 35040 İzmir, Türkiye

**Corresponding author (A. Erdem):* arzum.erdem@ege.edu.tr

Chemicals and apparatus

Electrochemical measurements were conducted using an AUTOLAB PGSTAT204 potentiostat controlled by NOVA 2.1.7 software (Eco Chemie, The Netherlands). A conventional three-electrode configuration was employed, consisting of a disposable pencil graphite electrode (PGE) as the working electrode, a platinum wire as the counter electrode, and an Ag/AgCl (3 M KCl) reference electrode (BAS, Model RE-5B, W. Lafayette, USA). All measurements were carried out inside a Faraday cage.

The S1 protein-specific optimized aptamer was obtained from Aptamer Group (UK), which developed, optimized, and holds the patent for this sequence targeting the SARS-CoV-2 S1 protein. According to the manufacturer's recommendations, the aptamer should be prepared using a suitable binding buffer. SARS-CoV-2 S1 protein was purchased from Sino Biological. A stock solution of the protein was prepared in ultra-pure water at a concentration of 250 µg/mL and stored at -80 °C until use. Working solutions of the S1 protein were prepared by dilution in phosphate-buffered saline (PBS, 50 mM, pH 7.40). MERS-CoV-S1 (MERS) protein was obtained from Sino Biological. A stock solution was prepared in ultra-pure water at a concentration of 150 µg/mL and stored at -80°C until use. Working solutions were prepared by dilution in phosphate-buffered saline (PBS, 50mM, pH 7.40). Influenza Hemagglutinin (HA) peptide was purchased from Sigma-Aldrich. A stock solution of HA was prepared in ultra-pure water at a concentration of 5 mg/mL and stored at -20°C. Diluted HA solutions were also prepared in PBS (50mM, pH 7.40). N-(3-Dimethylaminopropyl)-N′-ethylcarbodiimide hydrochloride (EDC) and N-Hydroxysuccinimide (NHS) were obtained from Sigma-Aldrich. All chemicals used in this study were of analytical reagent grade and purchased from commercial suppliers. Milli-Q grade ultra-pure water was used to freshly prepare all aqueous solutions.

Table S1. Average current values, % RSD values (n=3) and % change values obtained before/after S1P interaction with OPT at the electrode surface and in the solution phase.

	Interaction at electrode surface		Interaction in solution phase	
	**Average current value (μA), % RSD**	**% Change**	**Average current value (μA), % RSD**	**% Change**
**PGE**	195.38 ± 3.65<br>1.87%	--	195.38 ± 3.65<br>1.87%	--
**OPT control**	45.73 ± 19.26<br>42.10%	76.59% decrease	69,84 ± 7,42<br>10.63%	64.25% decrease
**OPT-S1P interaction**	41.02 ± 11.78<br>28.72%	10.31% decrease	60,68 ± 3,99<br>6.58%	13.12% decrease

* Interaction procedure at the electrode surface, average current value for S1 protein (S1P) control group was 208.67 ± 7.75 μA, % RSD= 3.71%, n=3. Interaction procedure in the solution phase, average current value for S1 protein (S1P) control group was 162.26 ± 6.79 μA, % RSD= 4.18%, n=3.

Table S2. Average current values, % RSD values (n=3) and % change values obtained before/after S1P interaction with OPT using different concentrations of redox probe solution.

	2.5 mM Redox Probe Solution		5 mM Redox Probe Solution	
	**Average current value (μA), % RSD**	**% Change**	**Average current value (μA), % RSD**	**% Change**
**PGE**	195.38 ± 3.65,<br>1.87%	--	311.99 ± 10.66,<br>3.42%	--
**OPT control**	69.84 ± 7.42,<br>10.63%	64.25% decrease	135.38 ± 3.18,<br>2.35%	56.61% decrease
**OPT-S1P interaction**	60.68 ± 3.99,<br>6.58%	13.12% decrease	116.32 ± 5.72,<br>4.91%	14.08% decrease

* In the measurements performed with the S1 protein (S1P) control group using 2.5 mM redox probe solution, the average current value was 162.26 ± 6.79 μA, % RSD= 4.18% (n=3). In the measurements performed with the S1 protein (S1P) control group using 5 mM redox probe solution, the average current value was 208.72 ± 18.31 μA, % RSD= 8.77% (n = 3).

Table S3. Average current values, % RSD values (n=3) and % change values obtained before/after S1P interaction with OPT in the presence and absence of EDC/NHS solution prepared in different buffers.

	In the absence of EDC/NHS		In the presence of EDC/NHS prepared in PBS solution		In the presence of EDC/NHS prepared in MES solution	
	**Average current value (μA), % RSD**	**% Change**	**Average current value (μA), % RSD**	**% Change**	**Average current value (μA), % RSD**	**% Change**
**PGE**	311.99 ± 10.66, 3.42%	--	311.99 ± 10.66, 3.42%	--	311.99 ± 10.66, 3.42%	--
**PGE-EDC/NHS**	--	--	328.52 ± 39.21, 11.93%	5.30% increase	444.19 ± 14.36, 3.23%	42.37% increase
**OPT control**	135.38 ± 3.18, 2.35%	56.61% decrease	191.26 ± 8.46, 4.41%	41.57% decrease	191.88 ± 5.67, 2.95%	56.80% decrease
**OPT-S1P interaction**	116.32 ± 5.72, 4.91%	14.08% decrease	161.28 ± 18.77, 11.64%	15.98% decrease	160.72 ± 4.76, 2.96%	16.24% decrease

* In the measurements performed with S1 protein (S1P) control group in the absence of EDC/NHS, average current value was 208.72 ± 18.31 μA, % RSD= 8.77% (n=3). In the measurements performed with the S1 protein (S1P) control group in the presence of EDC/NHS prepared in PBS solution, average current value was 289.36 ± 16.99 μA, % RSD= 5.87% (n=3). In the measurements performed with the S1 protein (S1P) control group in the presence of EDC/NHS prepared in MES solution, average current value was 320.32 ± 6.42 μA, % RSD= 2.00% (n=3).

Table S4. Average current values and % RSD values (n=3) and % change values obtained before/after the interaction of OPT prepared at different concentrations with S1 protein.

	Average current value (μA), % RSD (OPT control)	Average current value (μA), % RSD (after interaction)	% Change compared to OPT control
**PGE-EDC/NHS**	444.19 ± 14.36, 3.23%	--	--
**0.003 nM OPT**	169.49 ± 13.48, 7.96%	152.48 ± 20.32, 13.32%	10.03% decrease
**0.03 nM OPT**	150,41 ± 2,35, 1.57%	143.37 ± 11.28, 7.87%	4.68% decrease
**0.3 nM OPT**	191.88 ± 5.67, 2.95%	160.72 ± 4.76, 2.96%	16.24% decrease
**3 nM OPT**	162.28 ± 12.87, 7.93%	151.69 ± 3.69, 2.43%	6.52% decrease
**30 nM OPT**	150.11 ± 12.99, 8.65%	141.28 ± 23.81, 16.85%	5.88% decrease

* In the measurements performed with S1 protein (S1P) control group, average current value was 320.32 ± 6.42 μA (n=3).

Table S5. Average current values, % RSD values (n=3) and % change values obtained before/after S1P interaction with OPT at different interaction times.

	**Average current value (μA), % RSD (OPT control)**	**Average current value (μA), % RSD (after interaction)**	**% Change compared to OPT control**
**PGE-EDC/NHS**	444.19 ± 14.36, 3.23%	--	--
**5 min interaction**	191.88 ± 5.67, 2.95%	160.72 ± 4.76, 2.96%	16.24% decrease
**15 min interaction**	176.01 ± 4.96, 2.82%	144.16 ± 4.13, 2.86%	18.10% decrease
**30 min interaction**	171.78 ± 15.49, 9.02%	152.25 ± 4.08, 2.68%	11.37% decrease

*The average current value for S1 protein (S1P) control group at 5 min interaction time was 320.32 ± 6.42 μA (n=3). The average current value for S1 protein (S1P) control group at 15 min interaction time was 322.46 ± 15.22 μA (n=3). The average current value for S1 protein (S1P) control group at 5 min interaction time was 367.95 ± 14.74 μA (n=3).

Table S6. Average current values, % RSD values (n=3) and % change values obtained before/after S1P interaction with OPT at different immobilization times.

	**Average current value (μA), % RSD (OPT control)**	**Average current value (μA), % RSD (after interaction)**	**% Change compared to OPT control**
**PGE-EDC/NHS**	444.19 ± 14.36, 3.23%	--	--
**15 min immobilization**	176.01 ± 4.96, 2.82%	144.16 ± 4.13, 2.86%	18.10% decrease
**30 min immobilization**	181.67 ± 1.56, 0.86%	119.23 ± 7.64, 6.41%	34.37% decrease
**60 min immobilization**	161.46 ± 8.38, 5.19%	132.86 ± 7.43, 5.59%	17.71% decrease

*After 15 min immobilization, average current value for S1 protein (S1P) control group was 322.46 ± 15.22 μA (n=3). After 30 min immobilization, average current value for S1 protein (S1P) control group was 300.20 ± 20.62 μA (n=3). After 30 min immobilization, average current value for S1 protein (S1P) control group was 362.59 ± 22.06 μA (n=3).

Table S7. Experimental parameters were studied using SARS-CoV-2 S1 specific optimized aptamer-based electrochemical biosensor and the optimized conditions were presented.

Experimental parameters	Conditions	Selected value
**Interaction procedure**	Interaction procedures in the solution phase and at the electrode surface	Interaction procedures in the solution phase
**Redox probe concentration**	2.5 mM ve 5 mM	5 mM
**EDC/NHS activation**	Procedure followed in the presence and absence of EDC/NHS	Procedure followed in the presence of EDC/NHS
**Solution for the preparation of EDC/NHS**	PBS ve MES	MES
**OPT concentration**	0.003 / 0.03 / 0.3 / 3 / 30 nM OPT	0.3 nM OPT
**Interaction time**	5 / 15 / 30 min	15 min
**Immobilization time**	15 / 30 / 60 min	30 min

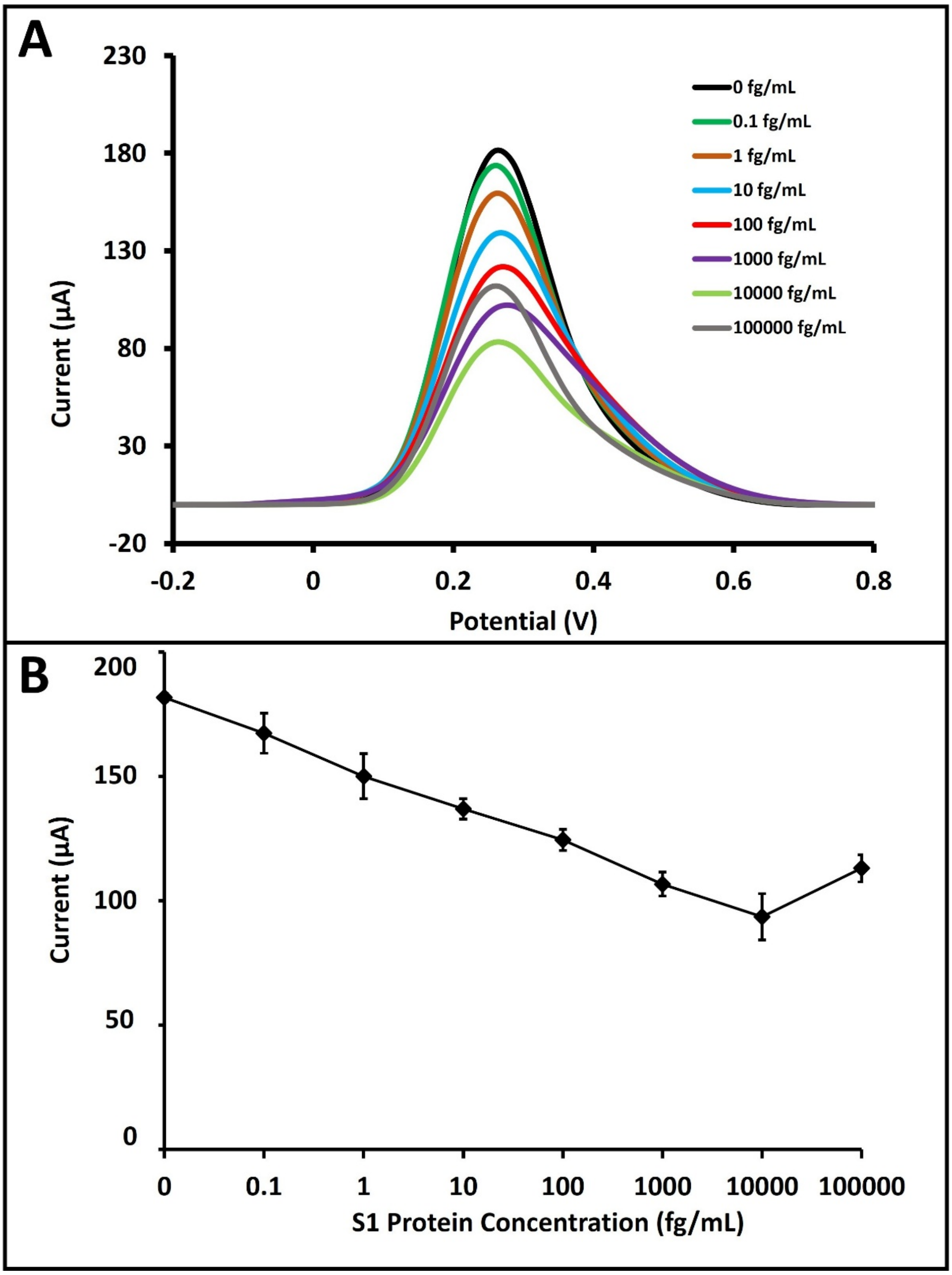

Fig S1. (A) DPV voltammograms and (B) line graph showing the average current values (n = 3) obtained after measurements in 5 mM redox solution across the S1 protein concentration range of 0–100,000 fg/mL in buffer medium.

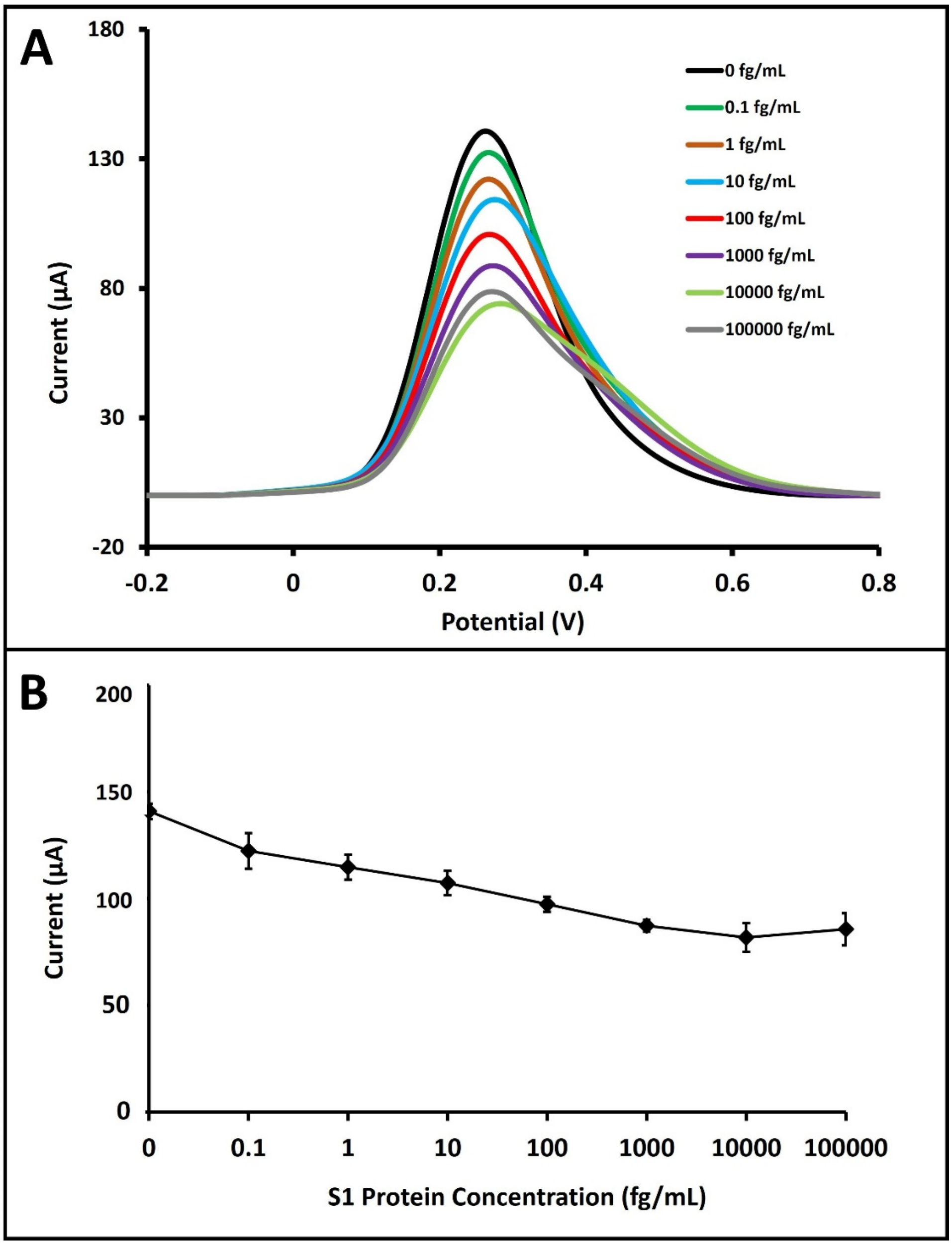

Fig S2. (A) DPV voltammograms and (B) line graph showing the average current values (n = 3) obtained after measurements in 5 mM redox solution over the S1 protein concentration range of 0–100,000 fg/mL in artificial saliva medium.

Table S8. Current responses and percentage changes relative to the control group obtained before and after the interaction of aptamer with 10 fg/mL S1 protein in artificial saliva at different dilution ratios.

**Dilution ratio**	**Before interaction with 10 fg/mL S1 protein (I, n=3) and RSD% (n=3)**	**After interaction with 10 fg/mL S1 protein (I, n=3) and RSD% (n=3)**	**Change**
1:10	119.26 ± 37.76<br>31.66%	94.46 ± 11.09<br>11.74%	20.79% decrease
1:20	140.87 ± 3.63<br>2.58%	107.27 ± 5.65<br>5.27%	23.85% decrease
1:50	108.72 ± 19.52<br>17.96%	103.18 ± 5.23<br>5.07%	5.10% decrease

Table S9. Relative error values of the developed optimer-based electrochemical biosensor for S1 protein detection in artificial saliva.

**Sample (fg/mL)**	**Found (fg/mL)**	**Relative error (%)**	**RSD (%) (n=3)**
0.1	0.116	15.58	6.78
1	0.911	8.87	5.10
100	107.732	7.73	3.63

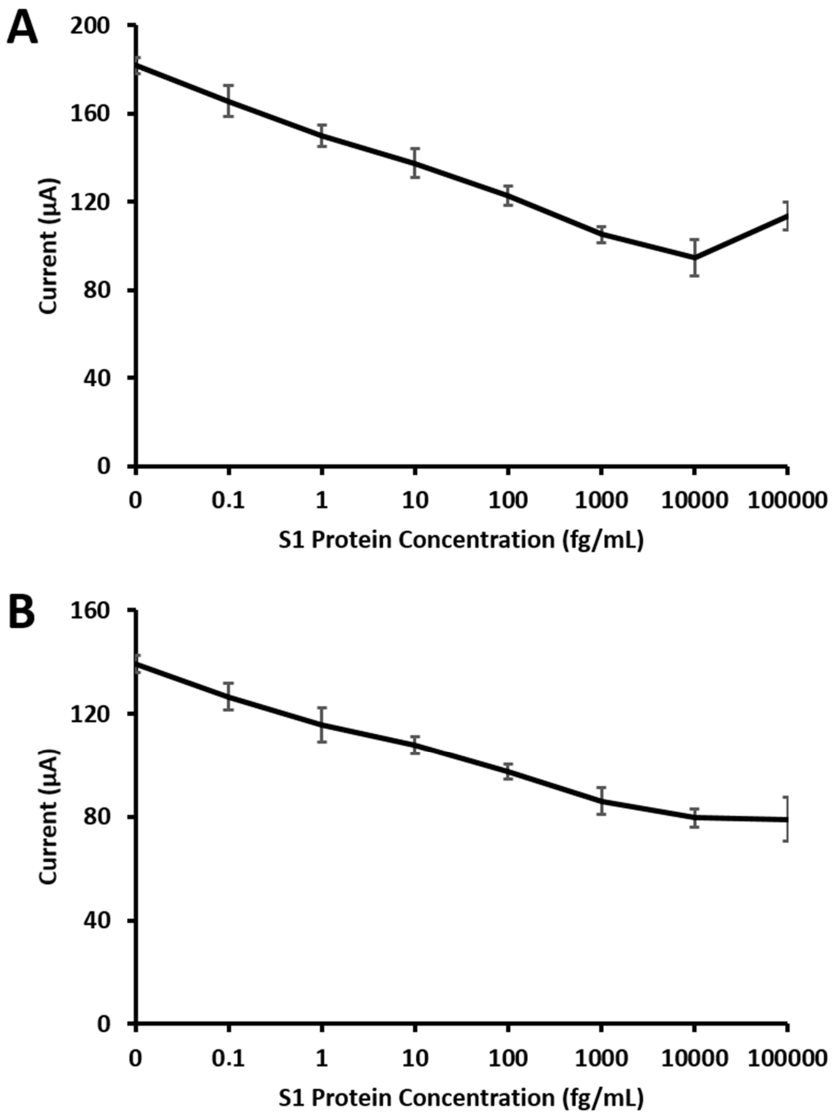

Fig S3. Studies on the determination of S1 protein using a smartphone-integrated potentiostat. (A) and (B) present the line graphs (n=3) obtained after DPV measurements in buffer and 1:20 diluted artificial saliva media, respectively, within the concentration range of 0.1 fg/mL to 100000 fg/mL.

Table S10. Results showing the relative error values of the S1 protein-specific optimer-based electrochemical biosensor developed for the detection of S1 protein in artificial saliva with a smartphone-integrated portable potentiostat.

Sample (fg/mL)	Found (fg/mL)	Relative error (%)	RSD (%) (n=3)
0.1	0.089	10.98	4.08
10	8.144	18.56	3.00
100	100.000	0.00	2.95
